# Fucose Ameliorates Tryptophan Metabolism and Behavioral Abnormalities in a Mouse Model of Chronic Colitis

**DOI:** 10.3390/nu12020445

**Published:** 2020-02-11

**Authors:** Mariya A. Borisova, Olga A. Snytnikova, Ekaterina A. Litvinova, Kseniya M. Achasova, Tatiana I. Babochkina, Alexey V. Pindyurin, Yuri P. Tsentalovich, Elena N. Kozhevnikova

**Affiliations:** 1 The Federal Research Center Institute of Cytology and Genetics of The Siberian Branch of the Russian Academy of Sciences, 630090 Novosibirsk, Russia; mariazolot@yandex.ru (M.A.B.); babochkinat@yahoo.com (T.I.B.); 2 International Tomography Center, The Siberian Branch of the Russian Academy of Sciences, 630090 Novosibirsk, Russia; koa@tomo.nsc.ru (O.A.S.); yura@tomo.nsc.ru (Y.P.T.); 3 Novosibirsk State University, Department of natural sciences, 630090 Novosibirsk, Russia; 4 Scientific Research Institute of Physiology and Basic Medicine, 630117 Novosibirsk, Russia; litvinovaea@physiol.ru (E.A.L.); achasovaks707@gmail.com (K.M.A.); 5 Siberian Federal Scientific Centre of Agro-BioTechnologies of the Russian Academy of Sciences, Krasnoobsk, 630501 Novosibirsk, Russia; 6 Institute of Molecular and Cellular Biology, The Siberian Branch of the Russian Academy of Sciences, 630090 Novosibirsk, Russia; aleksey.pindyurin@gmail.com

**Keywords:** DSS-induced colitis, inflammation, microbiota, odor preference, fucose, tryptophan

## Abstract

Growing evidence suggests that intestinal mucosa homeostasis impacts immunity, metabolism, the Central Nervous System (CNS), and behavior. Here, we investigated the effect of the monosaccharide fucose on inflammation, metabolism, intestinal microbiota, and social behavior in the Dextran Sulfate Sodium (DSS)-induced chronic colitis mouse model. Our data show that chronic colitis is accompanied by the decrease of the serum tryptophan level and the depletion of the intestinal microbiota, specifically tryptophan-producing *E. coli* and *Bifidobacterium*. These changes are associated with defects in the male mouse social behavior such as a lack of preference towards female bedding in an odor preference test. The addition of fucose to the test animals’ diet altered the bacterial community, increased the abundance of tryptophan-producing *E. coli*, normalized blood tryptophan levels, and ameliorated social behavior deficits. At the same time, we observed no ameliorating effect of fucose on colon morphology and colitis. Our results suggest a possible mechanism by which intestinal inflammation affects social behavior in male mice. We propose fucose as a promising prebiotic, since it creates a favorable environment for the beneficial bacteria that promote normalization of serum tryptophan level and amelioration of the behavioral abnormalities in the odor preference test.

## 1. Introduction

Inflammatory Bowel Disease (IBD) is a growing problem in the Western world, affecting up to 0.5% of the general population [1]. IBD includes Ulcerative Colitis (UC) and Crohn’s Disease (CD), both of which are idiopathic, chronic, and relapsing inflammatory disorders [2]. One of the UC animal models is based on a Dextran Sulfate Sodium (DSS) administration in drinking water. The mechanism of DSS action involves binding to medium-chain-length fatty acids present in the mouse colon, leading to the disruption of the epithelial barrier and intestinal inflammation [3]. A DSS-induced chronic colitis mouse model was previously reported to recapitulate a number of key processes characteristic to the clinical manifestations of such complex disease as IBD [4]. These include diarrhea, weight loss, intestinal inflammation, immune cells infiltration, and high pro-inflammatory cytokine expression levels during the acute phase of the developing colitis, intestinal barrier disruption, changes in the microflora composition, and some aspects of neurological disorders [5,6,7,8,9]. It has been shown that pro- and anti-inflammatory cytokines play a major role in the development of IBD. For instance, CD is associated with a Th1-type immune response, whereas UC is considered to be linked to a Th2-type immune response. At the same time, both CD and UC are associated with high levels of Th17 cytokines that induce the production of TNF-α, IL-1β, and chemokines [10]. Activation of TNF-α, IL-1β, and TGF-β induces necroptosis, whereas neuroinflammation is involved in amyloidogenesis, memory impairment, and multiple sclerosis [11,12,13]. Therefore, DSS-induced colitis serves as a great model to study complex interactions between microbes and the host in a state of inflammation, as well as provides a platform to test molecules capable of modulating microbiome-to-host crosstalk.

It has been proposed that fucose-containing molecules, like fucoidan, have an ameliorating effect on DSS-induced colitis [14], with fucose being the monosaccharide responsible for the anti-inflammatory effect [15]. Nearly 80% of the proteoglycan protective sheath in the intestine is composed of the mucin-2 polysaccharide side chains, where fucose is one of the two most exposed monosaccharaides due to its terminal position. For bacteria, it can function as an energy source, an adhesion site, or a virulence factor [16,17]. Host glycosylation is induced by the normal resident bacteria, and it is used for protection against infection and inflammation [18]. Thus, it would be of particular interest to test whether exogenous fucose attenuates the effects of DSS treatment on different aspects of chronic colitis.

One of the well-known IBD aspects well recapitulated in DSS-induced colitis model is the alteration of the host behavior. Anxiety-like behavior, decreased locomotion, reduced social interaction, alterations in expression of neuropeptide Y, and Brain Derived Neurotrophic Factor (BDNF) were reported upon DSS treatment in mice [5,6,19,20]. However, possible pathways underlying the gut-brain interactions are still discussed. In some studies, the connection between intestinal microbiota and behavior was well established via the identification of key metabolites responsible for the gut-brain crosstalk [21,22]. Changes in certain metabolites might also explain the behavioral features of the DSS-induced model, since DSS treatment has been shown to affect the host metabolism [23,24]. The aim of the present study was to investigate the effect of chronic colitis on major serum metabolites, to determine its physiological effect on the host behavior, and to evaluate the potential ameliorating effect of fucose on these aspects of IBD. Here, we demonstrate that the decrease of serum tryptophan during chronic DSS-induced colitis is accompanied by the depletion of the intestinal *E. coli* and *Bifidobacterium* strains, which are potential tryptophan producers. We further show that the lack of tryptophan results in the social behavior abnormalities associated with the insufficiency of the tryptophan metabolism. Finally, we demonstrate that the addition of fucose to the animals’ diet partially normalizes intestinal microflora, restores serum tryptophan, and rescues the behavioral phenotype.

## 2. Materials and Methods

### 2.1. Animal Housing

The study was conducted using the equipment of the Center for Genetic Resources of Laboratory Animals at the Institute of Cytology and Genetics of the Siberian Branch of the Russian Academy of Sciences (ICG SB RAS), supported by the Ministry of Education and Science of the Russian Federation (Unique identifier of the project RFMEFI62117X0015). All procedures were conducted under Russian legislation according to Good Laboratory Practice standards (directive #267 from 19.06.2003 of the Ministry of Health of the Russian Federation), inter-institutional bioethical committee guidelines, and the European Convention for the protection of vertebrate animals used for experimental and other scientific purposes; all procedures were approved by the inter-institutional bioethical committee, protocol #18.4 (14.10.2013).

All animals used had specific pathogen free (SPF) status, which was tested quarterly according to Federation of European Laboratory Animal Science Associations (FELASA) recommendations [25]. All experimental groups comprised 12–14 week-old male C57Bl/6 mice due to the widespread use of this genetic background in genome engineering. Therefore, the results obtained in this study could be applicable to transgenic animal models. We used 12–14 week-old female and male BALB/c mice to collect bedding for the olfactory preference test and to induce social and sexual experience in the test males.

All animals were weaned at three weeks of age and kept in groups of the same-sex siblings in open cages until the age of 10–12 weeks, and then placed in individually ventilated cages (Optimice, AnimalCare Systems). All animals were housed under 14 h/10 h light/dark photoperiod (light off at 16:00 h) with 22–24 °C temperature, 30–60% humidity, and 14–16 volumes of air exchange per hour; food (SSniff, Soest, Germany) and water were provided ad libitum. Individually ventilated cages were supplied with birch sawdust as litter and paper cups as shelter. Cages were replaced every seven days, except for during the 24 h before the behavioral tests. All behavioral tests were performed during dark period of time between 16:00 and 20:00 h by the same two observers. Animals were moved from the home cage to the experimental arena by capturing by the tail.

### 2.2. Chronic DSS Treatment

**Figure 1:**
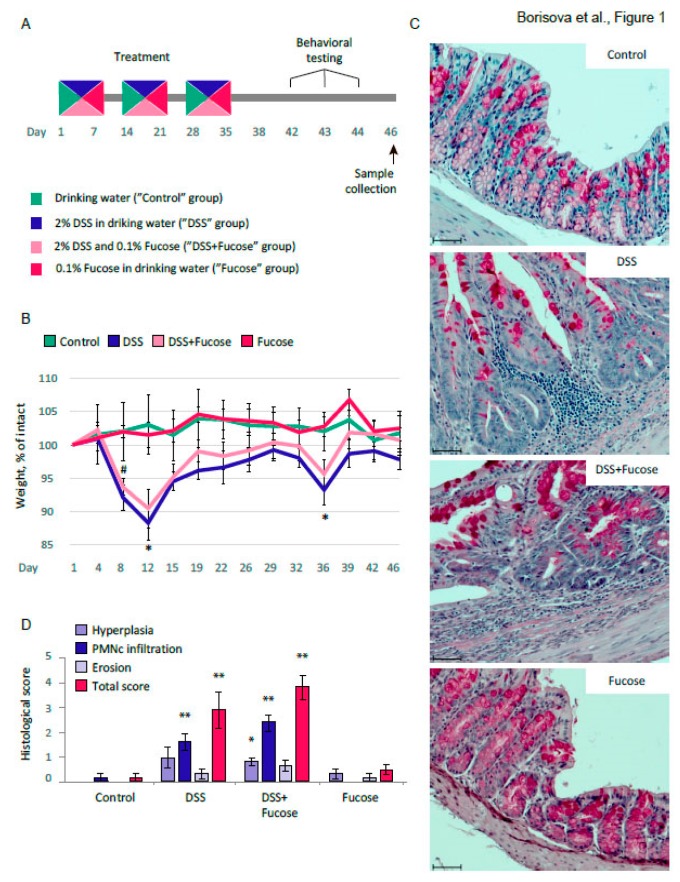
Fucose does not improve inflammatory response to chronic DSS treatment. (**A**) Chronic DSS treatment experimental design; (**B**) Body weight dynamics during chronic DSS-induced colitis. * = *p* < 0.05, “DSS” vs. “Control”, # = *p* < 0.05, “DSS + Fucose” vs. “Control”, Student’s *t*-test for independent samples. (**C**) PAS-stained histological sections of descending colon (scale bar 50 μm). (**D**) Histological scoring of the inflammatory response. * = *p* < 0.05, ** = *p* < 0.01, vs. “Control”, Mann-Whitney *u*-test).

The control group (“Control”, *n* = 15) received regular drinking water, “DSS” group (*n* = 15) received 2% DSS (NeoFroxx, Einhausen, Germany) in drinking water, “DSS + Fucose” group (*n* = 14) received 2% DSS in water supplemented with 0.1% fucose (Carbosynth, San Diego, CA, USA), and the “Fucose” group (*n* = 13) consumed 0.1% fucose in drinking water. The concentration of fucose used in this study is relevant to its physiological level in the mammalian intestine [17]. Animals received the above treatments for seven days, after which all drinking solutions were replaced by drinking water for another seven days. The cycle was repeated three times, making six weeks of chronic DSS treatment in total (42 days), after which the behavioral tests were conducted on the days 42–44. The experimental scheme is shown on Figure 1A. The animals were weighted twice a week during DSS treatment and given social and sexual experience. Chronic DSS treatment induced loose bloody stool, ruffled and unkempt fur. On the day 46 blood samples for Flow Cytometry (FC) were collected by retro-orbital sinus puncture, then the animals were decapitated and blood samples for nuclear magnetic resonance (NMR) spectroscopy were collected. Colon samples were placed in 10% neutral formalin for histological analysis or frozen in liquid nitrogen for gene expression measurements; fecal samples were taken and stored at −20 °C until further analysis.

### 2.3. Behavioral Testing

The order of the behavioral tests was the following: open field (OF) on day 42, smell test (ST) on day 43, and social olfactory preference test (OPT) on day 44 for the groups “Control”, “DSS”, “DSS + Fucose”, and “Fucose”. The OF was performed in a 1-m-wide circular arena for 5 min. Animal movements were recorded by the Ethostudio software [26], which calculated the distance walked by an animal (motor activity), area explored (exploratory activity) and time in the center (anxiety measure). The design of ST was the following: the test animals were food-deprived for 18 h prior to the test. Two tea infusers (Ikea, art. #469.568.00) containing either food pellets or plastic beads of a similar shape and color were placed into the test animal’s cage for 5 min. The time spent sniffing each infuser was recorded and analyzed manually. Only sniffing activity with the characteristic nose and whisker movements was scored as “sniffing” [27]. The OPT was designed similarly: two tea infusers were filled either with BALB/c female or male bedding and placed into the test animal’s cage for 5 min. Soiled bedding from BALB/c animals used in the test was taken from cages unchanged for a week before. The results of the ST and OPT are given as (time spent sniffing a specimen)/(total sniffing time) and expressed as percentages.

### 2.4. Social and Sexual Experience

It is known that previous social experience affects the maturation of neuronal circuits, involved in the regulation of social behavior in mice [28], and influences the subsequent social behavioral tests’ results [29]. The behavior of the sexually experienced male mice during the social odor preference tests differs from that of the naïve male mice [30]. To ensure the reproducibility of the behavioral tests, we used sexually and socially experienced male mice. This involved contacts with non-sibling animals of both sexes. As for the sexual experience, each test male was placed in the individual cage and co-housed with a BALB/c female mouse on the days 22 to 28 and from day 36 to day 39 (on the days free of DSS treatment). To obtain the social experience, an unfamiliar BALB/c male intruder was placed into a home cage of a C57Bl/6 male for 30 min [28] on day 30; the resident male was placed into a new cage upon obtainment of the social experience. As for behavioral testing upon the antibiotic treatment, C57Bl/6 male mice (“Rifampicin” group) aged 10 weeks were placed into individual cages (*n* = 15). Each male was co-housed with a BALB/c female mouse for 20 days, then the females were removed, and test males were given social experience with an unfamiliar BALB/c male intruder for 30 min. After that, the resident male was placed into a new cage.

### 2.5. Rifampicin Treatment

Socially and sexually experienced C57Bl/6 male mice (*n* = 15) received rifampicin solution in drinking water (0.075 mg/mL, Belmedpreparaty, Minsk, Belarus) for 13 days. After 7 days of the treatment, the behavioral tests were conducted in the following order: OPT (day 8), OF (day 12) and ST (day 13). On day 14, the animals were euthanized. Fecal samples were collected before the rifampicin treatment and on day 8.

### 2.6. 5-Hydroxytryptophan (5-HTP) Rescue Experiment

Male C57Bl/6 mice received chronic DSS treatment as well as social and sexual experience, as described above. The behavioral tests started on the day 42. 1.5 h before each test “DSS + 5-HTP” males (*n* = 14) were intraperitoneally injected with 5-HTP (40 mg/kg body weight, Sigma) in Phosphate-Buffered Saline (PBS), whereas “DSS” males were injected with PBS. On day 45, the animals were euthanized.

### 2.7. Histology

Colons were fixed in 10% neutral buffered formalin and embedded in paraffin. Paraffin sections (4 µm) were stained with Periodic acid-Schiff (PAS) stain (BioVitrum, Saint Petersburg, Russia) to examine general morphology and to detect goblet cell secretion. Azur-II-eosin stain was used to detect inflammatory cell infiltration. The sections were examined in a blinded manner. Images were taken with an AxioImager.M2 microscope using an Axiocam 305 color camera (Zeiss, Oberkochen, Germany). The number of epithelial cells in a crypt and the percentage of area involved in erosion were counted in PAS stained sections. Hyperplasia was defined as the percentage of cells per crypt above the mean number of those counted in the control sections [31]. Erosion was defined as the area of colonic epithelium where the entire crypt structure was lost and expressed as the percentage per section [32]. Polymorphonuclear cells (PMN cells, eosinophiles, and neutrophiles) were counted per section (x1000 magnification) in azur-II-eosin stained sections to evaluate inflammation severity [31,32,33]. The severity of histological injury was measured according to the scoring system described by Bergstrom with colleagues [31] and Ichikawa with colleagues [32] with modifications as follows:

- Hyperplasia above the control (0: <10%; 1: 10–50%; 2: 51–100%; 3: >100%),
- PMN cell infiltration (0: none, 1: mild, 2: moderate, 3: severe),
- Erosion (percentage of area involved) (0: <1%, 1: 1–15%, 2: 16–30%, 3: 31–45%, 4: 46–100%).

The maximum score that could result from this scoring (Total score) was 10.

### 2.8. Real-Time PCR

To measure the expression level of genes *Tnf-alpha*, *Il-1beta*, and *Ido1*, we purified total RNA from colonic samples using TRIzol reagent (Invitrogen, Waltham, MA, USA) according to the manufacturer’s recommendations. RNA concentration was measured with a NanoDrop 2000 spectrophotometer (ThermoScientific, Waltham, MA, USA). 1 µg of RNA was used in reverse transcription reaction, cDNA synthesis was performed using M-MuLV reverse transcriptase (SibEnzyme, Novosibirsk, Russia) according to the manufacturer’s recommendations. A mix of random hexa-deoxyribonucleotide and Oligo-dT primers were used for reverse transcription, after the completion of the reaction its volume of 20 µL was diluted to 100 µL with deionized water. Real-time PCR reaction was prepared using a BioMaster HS-qPCR SYBR Blue (2x) (BioLabMix, Novosibirsk, Russia), 5 µL of cDNA, and 250 nM specific primers. Amplification and detection were performed using a CFX96 Touch™ Real-Time PCR Detection System (BioRad, Hercules, CA, USA). Gene expression was normalized to *Tubb5* (*tubulin, beta 5 class I*) mRNA level as ∆Ct = 2 ^ (Ct_Tubb5_ mRNA − Ct_gene of interest_ mRNA). Primer sequences used for real-time PCR analyses are shown in Table S1.

DNA was purified form fecal pellets using a QIAamp DNA Stool Mini Kit (Qiagen, Hilden, Germany) according to the manufacturer’s recommendations. Real-time PCR of the murine *Enterococcus* species was performed using a commercial kit (Cat. #ESPP96S, BBT-LAB, Russia). To measure the abundance of tryptophan-producing bacteria, *E. coli, Bacteroides, Bifidobacterium,* and the 16S rRNA universal region, we performed real-time PCR with a BioMaster HS-qPCR SYBR Blue (BioLabMix, Novosibirsk, Russia), 5 µL of fecal DNA, and 300 nM specific primers. The data was normalized to 16S rRNA as ∆Ct = 2 ^ (Ct_16S_ − Ct_bacterium of interest_) and shown as log_10_(∆Ct). To detect the potentially tryptophan-producing species of *E. coli*, *Bacteroides*, and *Bifidobacterium*, we first performed the literature analysis to identify species and strains that are common in mice. For these species and strains we downloaded the available tryptophan operon (*trp*) sequences from the Ensembl Bacteria database [34] and aligned them using UGENE software [35]. We chose primer sequences so that they were specific to *trp* operon of all species within one genus, but not to *trp* operon of other genera. *TrpD* gene within *trp* was found to be the most convenient for primer design. Due to the high nucleotide variability of *trp* operon sequence, it is possible that not all *trp*-containing species of each genus were detected with each of the corresponding *trpD*-specific primer pair.

Colony PCR was carried out as follows: each colony was probed with a dispensable 10 μL pipette tip, and cells were suspended in 10 μL of deionized water. Afterwards, 1 μL of the suspension was taken in a PCR reaction containing HS-qPCR SYBR Blue (BioLabMix, Russia) and 300 nM *E. coli trpD*-specific primers.

### 2.9. Metabolite Extraction and NMR Spectroscopy

Blood samples were collected by decapitation and kept at room temperature for 15 min, centrifuged at 3000 rpm for 15 min, and then serum samples were stored at −70 °C until analyzed. The extraction of metabolites from serum was performed by using a short sample preparation protocol earlier evaluated for quantitative NMR-based metabolomics [36]. Namely, 100 µL of ice-cold methanol (HPLC grade, Scharlau, Barcelona, Spain) and 100 µL of ice-cold chloroform (HPLC grade, Scharlau, Spain) were added to 100 µL of serum and vortexed for 30 s, kept on ice for 10 min, and incubated at −20 °C for 30 min. The mixtures were centrifuged at 12,000 rpm and at 4 °C for 30 min to pellet proteins. The top hydrophilic fraction was collected to fresh vials and lyophilized using vacuum concentrator.

Dried extracts were re-dissolved in 600 µL of D_2_O (99.9%, Cambridge Isotope Laboratories Inc., Tewksbury, MA, USA) containing 6 µM sodium 4,4-dimethyl-4-silapentane-1-sulfonate (Cambridge Isotope Laboratories Inc., Tewksbury, MA, USA) as an internal standard and 20 mM deuterated phosphate buffer to maintain pH 7.4. All ^1^H NMR measurements were carried out as described previously [37] with the use of the AVANCE III HD 700 MHz NMR spectrometer (Bruker BioSpin, Ettlingen, Germany). The baseline processing and integration were done using the MestReNova v12.0 software. The metabolite signal assignment was confirmed by the addition of authentic compounds into samples and using the Human Metabolome Database [38,39] and our own experience in the metabolomic profiling of animal and human tissues and biofluids [37,40,41,42,43]. The concentrations of metabolites in samples were calculated by the peak area integration respectively to the internal standard.

### 2.10. FC Analysis

Blood samples were collected by an orbital sinus puncture. Red blood cells were lysed with ammonium chloride buffer (0.15 M NH_4_Cl; 0.01 M NaHCO_3_; 0.001 M EDTA) for 10 min at room temperature and leukocytes were centrifuged at 1500 rpm for 5 min at 4 °C. Then white blood cells were washed twice with 2% Bovine Serum Albumin (BSA) in PBS, resuspended in staining buffer (1% BSA, 0.1% sodium azide in PBS) and diluted up to the concentration of 1000–1200 cells/μL. 250 μL of cell suspension were stained with FITC-CD19, PE-CD3ε, FITC-CD4, PE/Cy7-CD8a anti-mouse antibodies (BioLegend, San Diego, CA, USA) for 120 min at 4 °C in the dark, and the samples were analyzed using a Guava easyCyte Flow Cytometer (Merck Millipore, Darmstadt, Germany). For analysis, 25,000 lymphocytes were counted in each sample. The number of blood CD4^+^- and CD8^+^-cells was expressed as the ratio of CD4^+^- and CD8^+^-percentages of CD3^+^-cells.

### 2.11. Bacterial Culture

About 100 μL of the intestinal contents from C57Bl/6 animals were dissolved in 500 μL of Tryptic Soy Broth (TSB) medium, plated in serial dilutions on TSB agar plates, and incubated overnight at 37 °C. We observed four morphologically distinct colony types, and performed colony PCR with each colony type using *E. coli trpD*-specific primers. We received PCR product from one of the four colony types, which was used for PCR analysis with primers targeting the V4–V5 region of eubacterial 16S rRNA genes [44]. The PCR product was used for Sanger sequencing, and the resulting sequence was aligned and identified as a part of the *E. coli* 16S rRNA gene using the BLAST tool [45]. The identified *E. coli* colony was further cultured on TSB agar plates. In order to identify the antibiotics sensitivity, we tested this culture for over 20 antibiotics and found that it was sensitive to a number of polyketides, cefalosporins, penicillin-type antibiotics, and others, and chose rifampicin for the further in vivo work.

### 2.12. Statistics

The data were tested for normality using the Kolmogorov-Smirnov test. All data are presented as mean ± Standard Error of the Mean (SEM), except for bacterial abundance, where the actual values for each sample are shown. The principles used in Ethostudio software were described previously [26,46]. The body weight dynamics was analyzed using a two-way repeated measures ANOVA followed by Student’s *t*-test for independent samples as a post hoc test. The significance of the FC analysis was evaluated using a two-way ANOVA followed by the Student’s *t*-test for independent samples as a post hoc test. The histological scores and gene expression data (except *Ido1* gene expression) were analyzed using the Kruskall-Wallis test followed by the post hoc Mann-Whitney *u*-test. *Ido1* gene expression data were analyzed using the two-way ANOVA and the post hoc Student’s *t*-test for independent samples. As for metabolomic analysis, the Principle Component Analysis (PCA), the two-way ANOVA and the post hoc Student’s *t*-test for independent samples were performed. The results of the bacterial real-time PCR analysis were analyzed using the χ^2^ test, since in some samples certain bacterial species were beyond the detection limit. The results of the behavioral tests were analyzed using a two-way ANOVA, followed by the post hoc Student’s *t*-test. Odor preference comparisons within each experimental group (female vs. male or food vs. beads) were performed using Student’s *t*-test for dependent samples. Female odor preference comparisons between different experimental groups were performed using Student’s *t*-test for independent samples.

## 3. Results

### 3.1. Fucose Does Not Rescue Intestinal Inflammation Upon Chronic DSS Treatment

We first questioned whether fucose had any ameliorating effect on DSS-induced colitis, and assessed the weight loss, histological changes, pro-inflammatory cytokine expression, and immune cell quantity upon chronic DSS treatment. For the histology and mRNA expression analysis, the sample size was 6; for the weight counts and FC analysis, 9–10 animals per group were taken.

We observed a significant weight loss in “DSS” and “DSS + Fucose” groups. There was a significant effect of DSS on the body weight (repeated measures ANOVA, *p* < 0.001), a significant effect of the repetition (repeated measures ANOVA, *p* < 0.001) and a significant interaction of DSS and repetition (repeated measures ANOVA, *p* < 0.001). The addition of 0.1% fucose did not rescue the weight loss upon DSS treatment, since there was no statistically significant effect of fucose factor on the body weight during the experiment. The Student’s *t*-test showed a significant weight loss on day 8 (*p* = 0.029) upon chronic DSS + Fucose treatment, as well as on day 12 (*p* = 0.010) and day 36 (*p* = 0.027) upon chronic DSS treatment in comparison to “Control” (Figure 1B).

There was a profound inflammatory response in the descending colon upon DSS treatment (Figure 1C). This was manifested as elongated crypts and an elevated number of cells per crypt (hyperplasia), neutrophil and eosinophil (polymorphonuclear cell, PMNc) infiltration, and disruption of the epithelial structure (erosion). There was a significant effect of DSS on the colon morphology as evaluated with the Kruskal-Wallis test, involving: hyperplasia (*p* = 0.0047), PMNc infiltration, (*p* < 0.001), erosion (*p* = 0.0280), and total score (*p* < 0.001). However, there was no statistically significant effect of fucose. The development of the colonic inflammation upon chronic DSS treatment as compared to the “Control” group was further confirmed with the Mann-Whitney *u*-test (“DSS”: PMNc infiltration, *p* = 0.008, total score, *p* = 0.006; “DSS + Fucose”: hyperplasia, *p* = 0.020, PMNc infiltration, *p* = 0.0065, total score, *p* = 0.0082, Figure 1D).

**Figure 2:**
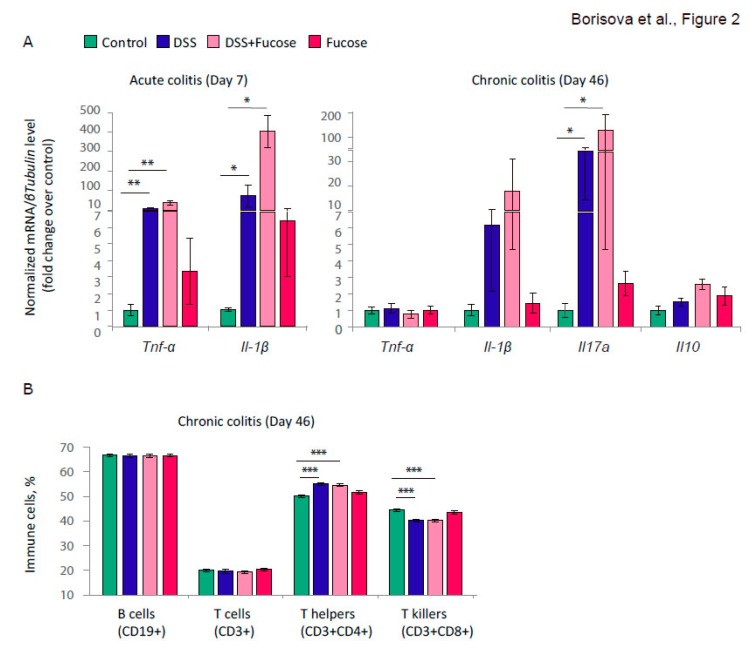
Fucose addition does not affect proinflammatory cytokine gene expression and blood immune cells. (**A**) Expression of cytokine genes in the descending colon normalized on the *βTubulin* (*Tubb5*) gene. * = *p* < 0.05, ** = *p* < 0.01, Mann-Whitney *u*-test. (**B**) Percentage of a given immune cell type. CD19+ and CD3+ are shown as percentages of lymphocytes, CD4+ and CD8+—as percentages of CD3+ lymphocytes. *** = *p* < 0.001, Student’s *t*-test for independent samples.

We have evaluated the observed inflammatory response with the gene expression analysis of characteristic proinflammatory cytokines *Tnf-α* and *Il-1β* during the acute phase of the inflammation (day 8) and during the chronic inflammation (day 46). We observed a strong up-regulation of both genes during the acute phase, but not upon chronic colitis. There was a significant effect of DSS on the proinflammatory cytokine gene expression as evaluated with the Kruskal-Wallis test: *Tnf-α*, *p* < 0.001, *Il-1β*, *p* < 0.001, Figure 2A. The Mann-Whitney *u*-test supported the observation that the acute DSS treatment induced a significant increase of the proinflammatory cytokine gene expression in the descending colon compared to the “Control” group (“DSS”: *TNF-α*, *p* = 0.006, *IL-1β*, *p* = 0.014: “DSS + Fucose”: *TNF-α*, *p* = 0.006, *IL-1β*, *p* = 0.012). We observed no statistically significant effect of fucose on the proinflammatory cytokine gene expression during acute colitis. Moreover, fucose did not affect the histological changes during the acute phase of the DSS-induced inflammation (Figure S1).

On day 46 of chronic DSS-induced colitis, there was a residual up-regulation of *Il-1β*, which was not statistically significant. There was a significant effect of DSS on *Il17a* proinflammatory cytokine gene expression (Kruskal-Wallis test: *p* < 0.001, Figure 2A). The Mann-Whitney *u*-test showed a significant increase of the *Il17a* gene expression in the descending colon as compared to the “Control” group (“DSS”: *p* = 0.014; “DSS + Fucose”: *p =* 0.014). However, there was no statistically significant effect of fucose on the *Il17a* gene expression during chronic colitis. Neither DSS nor fucose affected the expression of the anti-inflammatory *Il10* gene at this stage. The decrease in *Tnf-α* and *Il-1β* expression, elevation of *Il17a*, lack of *Il10* activation together with high histological scores suggested that the test animals were developing chronic inflammation.

To further investigate the effect of DSS and fucose on the systemic inflammation, we performed the FC analysis of blood immune cells. We have measured the percentage of T-cells (CD3+) and B-cells (CD19+) and the subpopulations of T-cells: T-helpers (CD3+ CD4+) and T-killers (CD3+ CD8+), since the changes in their ratio may indicate the ongoing systemic inflammation. FC did not reveal any effects of DSS or fucose on the percentage of CD3+ and CD19+ cells (Figure 2B). There was a strong effect of DSS treatment on the percentage of CD3+ CD4+ cells (two-way ANOVA, *p* < 0.001) and CD3+ CD8+ cells (two-way ANOVA, *p* < 0.001), but there was no effect of fucose on any cell type measured. Chronic DSS treatment increased the percentage of T-helper cells and decreased the percentage of T-killer cells in the blood of the test animals, meaning that the chronic inflammation was induced at the systemic level. In comparison to the “Control” group, the observed effects were statistically significant as revealed by the Student’s *t*-test (“DSS”: CD3+ CD4+, *p* < 0.001, CD3+ CD8+, *p* < 0.001; “DSS + Fucose”: CD3+ CD4+, *p* < 0.001, CD3+ CD8+, *p* < 0.001).

These results demonstrate that chronic inflammation was successfully induced by the DSS treatment; however, fucose had no ameliorating effect on any inflammatory parameters measured in our experiments.

### 3.2. Fucose Normalizes the Decrease of Blood Tryptophan Level upon Chronic DSS Treatment

**Figure 3:**
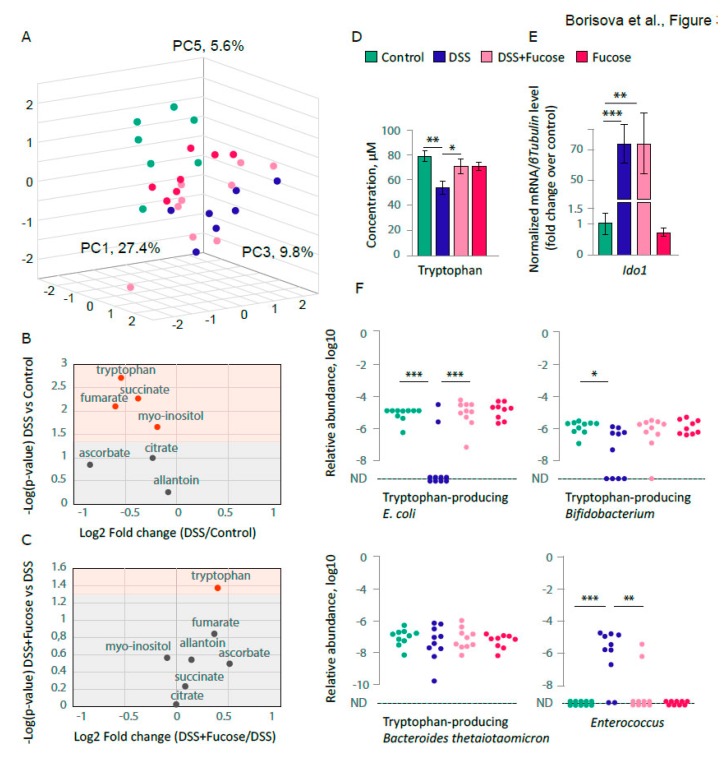
Fucose normalizes the levels of blood tryptophan and tryptophan-producing bacteria. (**A**) Metabolite concentrations in blood serum of test animals measured by NMR and analyzed using PCA plotted for PC1, PC3, and PC5. (**B**) Blood serum metabolite concentrations, “Control” vs. “DSS”, Student’s *t*-test for independent samples. Significant values (*p* < 0.05) are shown in red. (**C**) Blood serum metabolite concentrations, “DSS” vs. “DSS + Fucose”, Student’s *t*-test for independent samples. Significant values (*p* < 0.05) are shown in red. (**D**) Blood serum tryptophan concentration measured by NMR. * = *p* < 0.05, ** = *p* < 0.01, Student’s *t*-test for independent samples. (**E**) Expression of the *Ido1* gene in the descending colon normalized on the *βTubulin* (*Tubb5*) gene. ** = *p* < 0.01, *** = *p* < 0.001, Student’s *t*-test for independent samples. (**F**) Abundance of the intestinal bacteria measured in feces by real-time PCR normalized on the universal region of the *16S rRNA* gene, ND—not detected (* = *p* < 0.05, ** = *p* < 0.01, *** = *p* < 0.001, *χ*^2^ test).

In order to investigate the impact of chronic colitis on the host metabolism, we used the NMR spectroscopy-based quantitative analysis of blood serum metabolites (*n* = 7 for all groups except “DSS + Fucose”, where *n* = 9). This method allowed us to quantify 51 of the most abundant compounds. DSS treatment was associated with the overall change of the metabolic profile and with the significant changes in some metabolites. Principle Component Analysis (PCA) identified 29 principal components, nine of which described about 80% of metabolomic data variance. Two-way ANOVA revealed a significant interaction between DSS and fucose factors in PC1 (*p* = 0.046). There was a significant effect of DSS in PC3 (*p* < 0.001) and PC5 (*p* < 0.001), whereas a significant effect of fucose was found in PC3 (*p* = 0.023) and PC8 (*p* = 0.005). We plotted our samples using PC1, PC3, and PC5 (Figure 3A), which in total accounted for 42.78% of the NMR data variability.

To understand whether fucose had any ameliorating effect on metabolites upon chronic DSS treatment, we applied two-way ANOVA to search for a significant interaction between DSS and fucose factors for each of the identified metabolites. In this analysis, we found seven compounds: succinate, tryptophan, *myo*-inositol, allantoin, fumarate, citrate, and ascorbate. To test whether the DSS treatment itself had a significant effect on the serum levels of these metabolites, we applied Student’s *t*-test to compare “Control” and “DSS” groups (Figure 3B). We found that succinate (*p* = 0.006), tryptophan (*p* = 0.002), *myo*-inositol (*p* = 0.023), and fumarate (*p* = 0.008) were significantly reduced in chronic colitis. In order to investigate whether fucose normalized blood levels of these metabolites, we compared “DSS” and “DSS + Fucose” groups, and found that only tryptophan was significantly restored upon fucose addition during chronic DSS treatment (*p* = 0.042, Student’s *t*-test, Figure 3C). Chronic colitis resulted in about 30% loss of serum tryptophan, which was restored to the control level with fucose addition (Figure 3D). Since it has been previously shown that the serum tryptophan depletion during colitis is associated with the up-regulation of *indoleamine 2,3-dioxygenase 1 (Ido1)* gene expression, one of the enzymes that catalyze the rate-limiting step in the kynurenine pathway [47,48], we measured the expression level of this enzyme in the intestinal samples taken from the test animals. Two-way ANOVA revealed a significant effect of DSS on *Ido1* expression (*p* < 0.001) and, in agreement with the previous studies, chronic DSS treatment resulted in a significant up-regulation of the *Ido1* gene expression (*p* < 0.001, Student’s *t*-test, Figure 3E). However, the *Ido1* gene expression was the same in “DSS” and “DSS + Fucose” treated animals, indicating the absence of the effect of fucose on the Ido1-dependant tryptophan metabolism.

### 3.3. Fucose Rescues Depletion of the Intestinal E. coli upon Chronic DSS Treatment

We proposed that the metabolic changes described above resulted from significant microflora shifts that occur during chronic DSS colitis. Tryptophan is an essential amino acid that primarily comes from diet and can also be synthetized by some bacterial species. In order to investigate whether chronic DSS treatment affected the level of bacteria that could potentially synthetize tryptophan, we performed real-time PCR measurements of these bacterial strains in the fecal samples of the test animals (10 per group, except for “Fucose” (*n* = 9)). We used species-specific primers complementary to *trpD* gene, a part of the tryptophan metabolism operon (*trp*), to measure the bacterial strains capable of tryptophan synthesis. We found a significant reduction of *trpD*-containing *E. coli* and *Bifidobacterium* strains upon DSS treatment in comparison to the “Control” as evaluated with the *χ*^2^ test: *E. coli*, *p* < 0.001, *Bifidobacterium*, *p* = 0.025; however, there was no effect on *trpD*-containing *Bacteroides thetaiotaomicron* (Figure 3F). The addition of fucose rescued the effect of chronic DSS colitis on the abundance of *trpD*-containing *E. coli* (“DSS” vs. “DSS + fucose”, *p* < 0.001, *χ*^2^ test), but not *Bifidobacterium*. In order to evaluate whether our model reproduces previously reported increase of *Enterococcus* species in chronic DSS colitis [49], we measured *Enterococcus spp*. in the fecal samples of the test animals. In agreement with Berry and colleagues [49], we found that *Enterococcus* species strongly proliferated upon DSS treatment in comparison to the “Control” (*p* < 0.001, *χ*^2^ test). Moreover, we found that this effect was rescued by the addition of fucose, which rendered *Enterococcus* undetectable in most samples in the “DSS + Fucose” group (“DSS” vs. “DSS + Fucose”, *p* = 0.007, *χ*^2^ test).

These data demonstrate that fucose can specifically regulate different bacterial species and therefore might affect the host metabolism.

### 3.4. Fucose Rescues Lack of Social Odor Preference upon Chronic DSS Treatment in Male Mice

As we observed in the present work, chronic DSS treatment results in a substantial reduction of blood tryptophan, which is an essential amino acid and a precursor in the biosynthesis of monoamine serotonin and trace amine tryptamine, both involved in the regulation of animal behavior as neurotransmitters. Thus, we decided to test whether the reduction of tryptophan upon chronic colitis has an effect on mouse behavior. One of the widely used tests to evaluate the serotonin-dependent behavior in mice is the OPT where a test male is given a choice between a male and a female odor samples. Therefore, we used OPT to test the serotonin-dependent behavior, ST—to evaluate the general odor recognition, and OF—to measure the general activity (*n* = 13–15).

**Figure 4:**
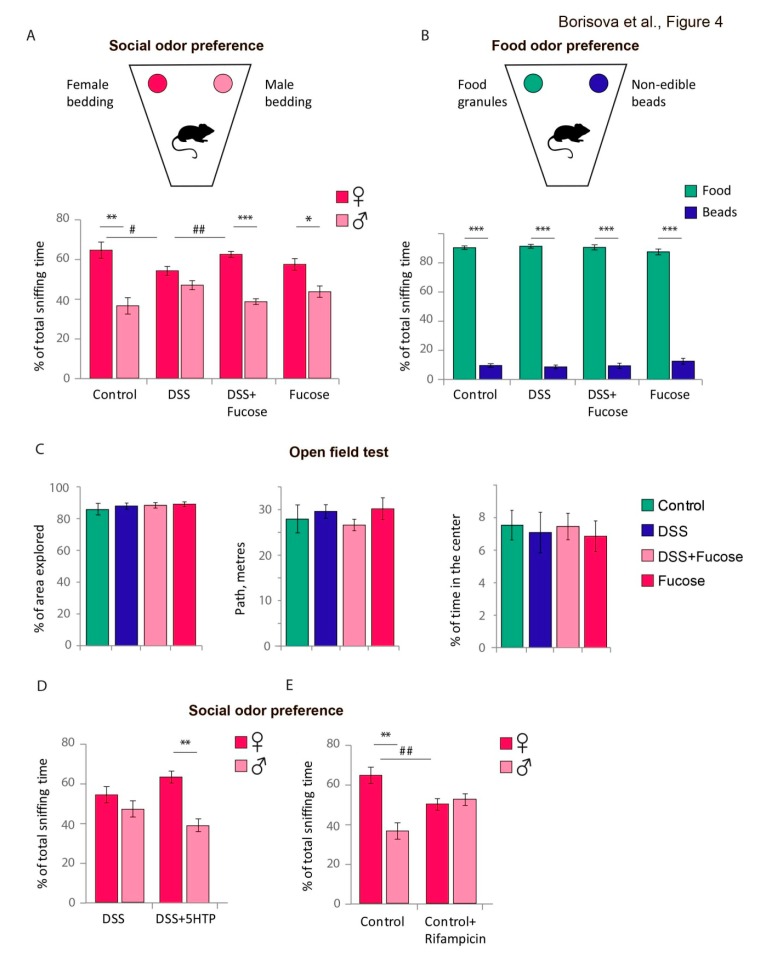
Chronic colitis affects female bedding preference by male mice in the OPT. (**A**) Total time an animal spent sniffing female or male bedding odor during the OPT. * = *p* < 0.05, ** = *p* < 0.01, *** = *p* < 0.001, Student’s *t*-test for dependent samples, # = *p* < 0.05, ## = *p* < 0.01, Student’s *t*-test for independent samples. (**B**) Total time an animal spent sniffing food or beads during the ST. *** = *p* < 0.001, Student’s *t*-test for dependent samples. (**C**) Exploratory activity (% of area explored), motor activity (path walked) and general anxiety (time spent in the center of the arena) in the OF test. (**D**) Total time an animal spent sniffing female or male bedding odor during the OPT after the injection of 5-HTP or PBS. ** = *p* < 0.01, Student’s *t*-test for dependent samples. (**E**) Total time an animal spent sniffing female or male bedding odor during the OPT with or without antibiotic treatment. ** = *p* < 0.01, Student’s *t*-test for dependent samples, ## = *p* < 0.01, Student’s *t*-test for independent samples.

In OPT, the control male mice normally prefer the female odor to male one [29,30]. Chronic DSS treatment abolished this preference, whereas the addition of fucose to DSS restored the normal female odor preference. Two-way ANOVA revealed a significant interaction between DSS and fucose factors for female odor preference (*p* = 0.010). The DSS-treated males spent less time sniffing the female sample than the control males (*p* = 0.034, Student’s *t*-test, Figure 4A). Fucose combined with DSS increased the female odor sniffing time as compared to DSS (“DSS” vs. “DSS + Fucose” *p* = 0.005; Student’s *t*-test). The control males strongly preferred the female bedding odor to the male bedding odor (*p* = 0.004, Student’s *t*-test, Figure 4A), whereas chronic DSS treatment resulted in the lack of a preference towards the female bedding odor in OPT (*p* = 0.13, Student’s *t*-test). The addition of fucose to DSS ameliorated the effect of chronic DSS treatment on sniffing behavior so that the “DSS + Fucose” male mice preferred the female bedding odor to the male bedding odor (*p* < 0.001, Student’s *t*-test). The “Fucose” mice also preferred the female odor to the male one (*p* = 0.036, Student’s *t*-test).

DSS treatment did not affect the general odor recognition, since all mice tested discriminated the food odor from the odorless beads of the same shape and color (Food vs. Beads, *p* < 0.001 for all groups, Student’s *t*-test, Figure 4B). There were no statistically significant differences in the OF test, and all animals exhibited normal motor and exploratory activity (Figure 4C).

In order to test the role of tryptophan metabolite-dependent neurotransmission in the behavioral phenotype described above, we performed the OPT in DSS-treated animals injected with serotonin precursor 5-HTP prior to the test. We found that 5-HTP treatment resulted in restored female odor preference in DSS-treated male mice (*p* = 0.006, Student’s *t*-test, Figure 4D), suggesting that the observed behavioral phenotype depends on the serotonin receptor neurotransmission. To further confirm the involvement of the previously described *trp*-containing bacteria, we used an antibiotic to deplete this strain in otherwise untreated C57Bl/6 male mice. Using bacterial culture methods, we found that this particular *E. coli* strain is sensitive to rifampicin. The rifampicin treatment of C57Bl/6 male mice resulted in the reduced female sniffing time as compared to the “Control” (*p* = 0.005, Student’s *t*-test, Figure 4E) and in the lack of the female odor preference in OPT (*p* = 0.67, Student’s *t*-test, Figure 4E), indicating that changes in microflora are a key to the behavioral phenotype observed in OPT upon chronic colitis.

## 4. Discussion

In the present study, we used DSS-induced chronic colitis mouse model to study the effect of chronic colitis on microflora, metabolism and behavior and to evaluate if fucose has potential to ameliorate any of these aspects.

First, we confirmed that our model of DSS-induced inflammation exhibited the features characteristic to chronic colitis. DSS treatment led to a substantial weight loss, profound inflammation and morphological changes in the descending colon, and a shift in the blood CD4+/CD8+ T-cell ratio (Figure 2B). We observed a strong overexpression of pro-inflammatory cytokines during the acute phase of the developing colitis accompanied by the elevated morphological scores in the intestine (Figure 1C and Figure S1). However, in the chronic state, we observed only residual changes in the gene expression of the pro-inflammatory cytokines as well as strong morphological defects in the intestine, indicating that chronic colitis successfully developed.

We further tested whether fucose—a terminal sugar in the polysaccharide chains of the host intestinal glycans is able to modulate the aspects of chronic inflammation in the animal model of DSS-induced colitis since host-derived fucosylated glycans create a landscape for a number of beneficial commensal microorganisms [50,51]. Some reports have already demonstrated the anti-inflammatory activity of fucoidan, fucoidan-containing extracts and free fucose in different experimental models in vitro and in vivo [15,52,53,54,55,56,57,58]. In contrast to the previous report [15], we were unable to detect any ameliorating effect of free fucose on the intestinal inflammation neither in the acute phase of colitis (Figure 2 and Figure S1) nor in its chronic form (Figure 1 and Figure 2). Since He and colleagues [15] did not mention the health status of the animals they used, this discrepancy can be attributed to the different microflora of the test mice. At the same time, He and co-authors generated a state of milder acute colitis, as can be judged from 3–4 fold overexpression of pro-inflammatory cytokine genes [15], versus 15–500-fold overexpression detected in the present study. Thus, it might be possible that in the case of severe colitis, fucose no longer retains its ameliorating effect on inflammation.

Interestingly, chronic DSS treatment also resulted in a substantial shift of the host metabolism as identified by NMR spectroscopy of the most abundant serum metabolites (Figure 3A). This finding confirms the systemic nature of chronic inflammation since blood metabolome reflects the major changes within the body including both host and microbiota metabolism. It has been previously shown that the acute and chronic DSS-treatment leads to a substantial change of the intestinal microflora [49,59,60], which is a result of the immune system activation, epithelial cell defense, and the host glycan landscape disruption [61,62,63,64]. At the same time, changes in the intestinal microbiota result in a different metabolic outcome and in the perturbation of the host metabolism. For instance, it has been noticed that the patients with acute and chronic forms of IBD have reduced tryptophan level in the blood plasma [65,66]. Moreover, the targeted analysis revealed that the plasma level of tryptophan significantly decreased in DSS-induced mouse model of acute colitis [47,48]. Our data support these findings, since we observe the impressive 30% reduction of serum tryptophan upon chronic DSS treatment. As there was no such a drastic reduction of other essential amino acids, we reasoned that the observed decrease of serum tryptophan was not a result of the reduced food intake or the impaired intestinal absorption.

It has been proposed that the tryptophan reduction during the inflammation is caused by the elevated activity of kynurenine pathway that leads to the tryptophan degradation in favor of nicotinamide adenine dinucleotide (NAD+). The key regulators of this process are the pro-inflammatory cytokines and indoleamine 2,3-dioxygenase 1 (IDO1), one of the enzymes that catalyze the rate-limiting step in the kynurenine pathway [47,48,66,67]. In agreement with these studies, we observed a strong up-regulation of the *Ido1* gene expression during chronic colitis (Figure 3E). However, we noticed that the addition of fucose during chronic colitis normalized serum tryptophan, but affected neither *Ido1* nor proinflammatory gene expression (Figure 2A and Figure 3D,E). These results suggest that there should be other mechanisms regulating tryptophan metabolism during the chronic inflammation. Further analysis of other metabolites of the kynurenine pathway including NAD+, which was not detected in our NMR spectra, would help to develop this hypothesis.

Given that commensal bacteria in the intestinal tract can synthesize substantial quantities of tryptophan [68,69], we hypothesized that chronic DSS treatment was associated with the decrease of tryptophan-producing intestinal bacteria. We measured the levels of some of tryptophan-producing bacteria that reside in the mouse intestinal tract and found a significant reduction of *E. coli* and *Bifidobacterium*. Moreover, the addition of fucose normalized the level of *E. coli* upon chronic colitis (Figure 3F), which correlates with the normalization of serum tryptophan. This finding supports our hypothesis that the reduction of serum tryptophan during chronic inflammation might be a result of the depletion of tryptophan-producing bacteria. Presumably, fucose creates a favorable environment for commensal *E. coli*, since the gavage of the same strain of cultured *E. coli* to the test mice during chronic DSS treatment did not result in the assimilation of these bacteria in the intestine (data not shown). This agrees with the idea that the proteoglycan landscape is essential for the creation of healthy microflora, and its disruption during the inflammation disintegrates the bacterial composition in the intestine. Our observation that the fucose treatment substantially reduced the number of *Enterococcus* species, which expanded upon chronic DSS treatment (Figure 3F) supports this hypothesis. It has been previously shown that *Enterococcus* species accumulate in chronic colitis [49], and the addition of fucosylated glycans during the infection abolished the overgrowth of *Enterococcus* bacteria [70]. So far, it is unclear whether there is an interrelation between the reduction of *E*. *coli* and the expansion of *Enterococcus*. A set of in vitro experiments is needed in order to understand the primary bacterial target of fucose and to elucidate the chain of events that lead to the reorganization of the microbial community upon fucose treatment.

Tryptophan reduction is thought to be at least partially responsible for the development of depression in patients with IBD. Tryptophan is a precursor in a rate-limiting step of serotonin synthesis, a neurotransmitter involved in the regulation of social behavior [71], and tryptamine—a neuromodulator, a trace amine-associated receptor 1, and serotonin receptor agonist [72]. Our data demonstrate that indeed, DSS-induced chronic colitis results in a disruption of socially-relevant behavior; the male mice after the chronic DSS treatment did not prefer the female to the male bedding odor (Figure 4A), whereas their smell preference towards the food odor and the general motor activity were unaffected (Figure 4B,C). These data agree with the previous reports showing that the male mice lacking brain serotonin exhibit no preference between male and female bedding [73]. Therefore, the lack of tryptophan could explain the social behavior phenotype upon chronic DSS colitis. In support of this hypothesis, the administration of a serotonin precursor (5-HTP) to animals with chronic DSS-colitis resulted in the rescue of the behavioral phenotype and restored female bedding preference in the test males in OPT.

Host intestinal microbes are believed to influence the anxiety and depressive-like behavior [74,75], stress response [76], and monoamine levels in the brain [77,78,79]. In order to test whether the reduction of *E. coli* capable of tryptophan production results in the behavioral phenotype described above, we used rifampicin treatment to deplete this bacterial strain. The rifampicin administration completely mimicked DSS chronic colitis in terms of the male mouse behavior in OPT so that male mice were unable to distinguish the male and female bedding (Figure 4E). This result supported our hypothesis that the inability of the test animals to discriminate the female and male bedding in OPT upon chronic DSS colitis is dependent on the intestinal *E*. *coli* availability and the serotonin receptor-dependent neurotransmission.

Interestingly, fucose rescued the effect of the chronic DSS treatment on the male mouse behavior in OPT and resulted in a significant increase of the female bedding sniffing time (Figure 4A). This result supports the idea that the observed behavioral changes are associated with the bacterial shifts in the intestine. It also suggests that fucose or its derivatives can be used as potential modulators of behavioral abnormalities observed in IBD. To our knowledge, there is at least one report that links fucose to mouse social behavior [80]. Park and colleagues demonstrated that the lack of fucose mutarotase (FucM), an enzyme that facilitates fucose incorporation into protein [81], resulted in the male-like sexual behaviors in female mice. Even though the authors did not consider a possible involvement of microflora in the phenotype, the intestinal bacteria and their metabolism might influence the behavioral outcome in FucM mutants. Thus, it would be interesting to investigate the effect of other monosaccharides present within mucin-2 proteoglycan on the microbiota and behavior.

There are multiple reports that investigate the ameliorating effect of oligosaccharides, sugars, and proteoglycans on inflammation [15,82,83,84,85,86]. However, there are just a few papers [87,88], that focus on their effect on other aspects of complex diseases such as UC and CD. In the present study, we demonstrated that the modulation of the host microflora by a monosaccharide fucose did not affect the severity of the chronic colitis in terms of the intestinal inflammatory response, but partially ameliorated the changes in metabolism and behavior. We suggest that it is worth testing the activity of drugs in every aspect of IBD, since their activity might be independent of the inflammation itself. At the same time, fucose and small molecules of similar action might provide a promising strategy to develop microflora- and metabolism-modifying drugs to target specific aspects of various diseases. Such compounds could broaden the range of clinical approaches and provide an economically attractive opportunity alternative to the prebiotic and probiotic system.

## Supplementary Materials

The following are available online at https://www.mdpi.com/2072-6643/12/2/445/s1, Figure S1: Fucose does not improve inflammatory response to acute DSS treatment, Table S1: Primers used for the study.
