# Supplementary material for: Fucose Ameliorates Tryptophan Metabolism and Behavioral Abnormalities in a Mouse Model of Chronic Colitis
Source: Nutrients. 2020 Feb 11;12(2):445. doi: 10.3390/nu12020445 (PMC7071335; doi:10.3390/nu12020445)
Supplement: Supplementary file 1 [file nutrients-12-00445-s001.pdf]

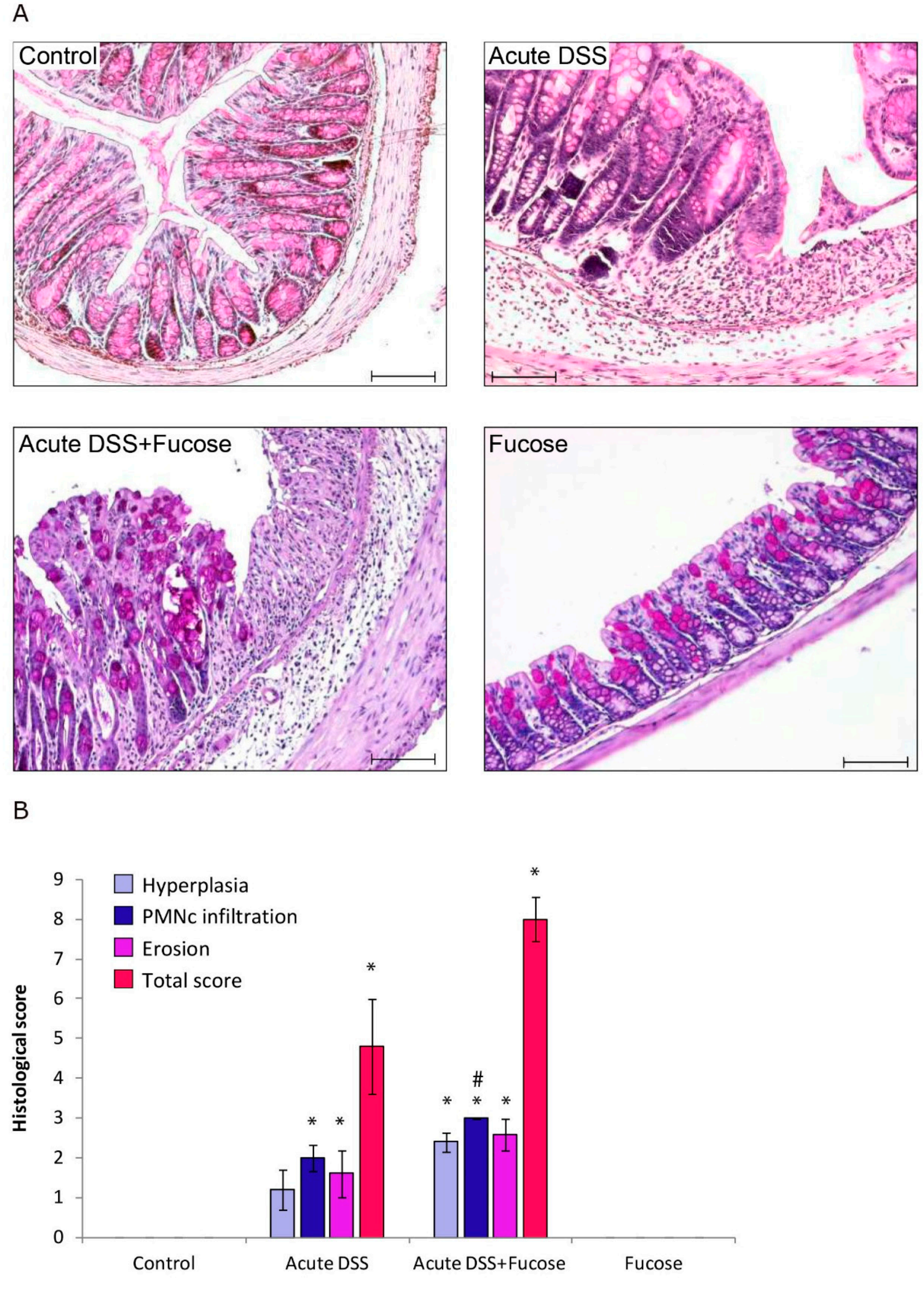

Figure S1. Fucose does not improve inflammatory response to acute DSS treatment. **A.** PAS-stained histological sections of the descending colon (scale bar 100 μm). **B**. Histological scoring of the inflammatory response. * = p<0.05 vs Control, # = p<0.05 vs "Acute DSS", Mann-Whitney U-test.

Table S1. Primers used for the study.

Target	Primer name	Primer sequence 5' -> 3'
Mouse *Tnf-α*	TNF_RNA F	CCCTCACACTCAGATCATCTTCT
	TNF_RNA R	GGCACCACTAGTTGGTTGTCTTT
Mouse *Il-1β*	Il1b-F	TGAAGTTGACGGACCCCAAA
	Il1b-R	TGATGTGCTGCTGCGAGATT
Mouse *Ido-1*	Ido-1 F	CGAGGGGATGACGATGTTCG
	Ido-1 R	CAGTCCCCACCAGGAAATGA
E.coli TrpD gene	ESCH_TRPD_F	ACCTGGCAGATCAGTTGC
	ESCH_TRPD_R	GAGAAAGCATCAGCACCG
Bifidobacterium TrpD gene	BIFI_TRPD_F	ATGGCCGA(A/T/G)ATCACATGG
	BIFI_TRPD_R	GTCCACAA(A/G)CCACTCCGATT
Mouse *Tubb5*	betaTub_F	TGAAGCCACAGGTGGCAAGTAT
	betaTub_R	CCAGACTGACCGAAAACGAAGT
16S rRNA universal region	16S_F	TCCTACGGGAGGCAGCAG
	16S_R	ATTACCGCGGCTGCTGG
Mouse *Il10*	Il10-F4	AAGGCAGTGGAGCAGGTGA
	Il10-R4	CTATGCAGTTGATGAAGATGTCA
Mouse *Il17a*	Il17a-F4	TCAAAGCTCAGCGTGTCCAA
	Il17a-R4	GGGTCTTCATTGCGGTGGA
